# Balancing efficiency and misinterpretation: general practitioners' perspectives on communicating diagnostic test results in the digital era

**DOI:** 10.1093/fampra/cmaf113

**Published:** 2026-01-13

**Authors:** Frederieke A M van der Mee, Chelsea B de Zeeuw, Jesse Jansen, Jochen W L Cals, Anneke N van Dijk-de Vries

**Affiliations:** Department of Family Medicine, Care and Public Health Research Institute, Maastricht University, P. Debyeplein 1, 6229 HA Maastricht, The Netherlands; Department of Family Medicine, Care and Public Health Research Institute, Maastricht University, P. Debyeplein 1, 6229 HA Maastricht, The Netherlands; Department of Family Medicine, Care and Public Health Research Institute, Maastricht University, P. Debyeplein 1, 6229 HA Maastricht, The Netherlands; Department of Family Medicine, Care and Public Health Research Institute, Maastricht University, P. Debyeplein 1, 6229 HA Maastricht, The Netherlands; Department of Family Medicine, Care and Public Health Research Institute, Maastricht University, P. Debyeplein 1, 6229 HA Maastricht, The Netherlands

**Keywords:** electronic health records, patient access to records, patient portal, health communication, general practice, general practitioners

## Abstract

**Background:**

Since patients increasingly have online access to their diagnostic test results, general practitioners (GPs) have reduced control over how this information is communicated. This shift introduces new challenges in communication and interaction with patients and requires a better understanding of how GPs experience and manage communication in an evolving digital healthcare landscape.

**Objectives:**

To explore GPs' experiences and perceived challenges in communicating information about diagnostic test results to patients in the context of increasing digital accessibility.

**Methods:**

In 2024, we conducted a qualitative study using semi-structured interviews with purposively sampled Dutch GPs in the Netherlands. Interviews were audio recorded, transcribed verbatim, and analyzed using thematic analysis. Key themes reflecting experiences and challenges related to the communication of diagnostic test results were identified.

**Results:**

Eighteen participants were interviewed in the study. Three overarching themes emerged from the data: (i) managing patient expectations; (ii) purpose-driven communication strategies; and (iii) balancing efficiency and patient engagement in communicating test results.

**Conclusions:**

GPs considered patients' online access to diagnostic test results a double-edged sword—while it may support more efficiency in the healthcare process, it also introduces communication challenges, particularly due to patients' misinterpretation of clinically insignificant findings and the use of medical jargon in reports. These findings highlight the need for tailored communication strategies and improvement of information provided in online patient portals.

Key messages

- GPs face new challenges as patients access their diagnostic test results online.
- Patients' online access limits GPs' control over test result communication.
- Managing patient expectations is crucial for effective GP–patient communication.
- Online access enhances but also reduces efficiency in result communication.
- Information in online portals must improve to support patient interpretation.

## Introduction

Patients worldwide increasingly access their medical data via online patient portals, including diagnostic test results ordered by their general practitioner (GP) [1–3]. While this development aims to enhance patients' knowledge, self-efficacy, engagement, and communication with health care providers [4–6], it also reduces the GP's control over how and when test results are communicated. Traditionally, GPs provide context, adjust messages to individual patients, and offer guidance during consultations [7]. With direct online access, however, patients increasingly rely on patient portals as their primary source of information and often view results without prior or accompanying explanation [8]. This may cause difficulties in interpreting the significance of abnormal findings within the broader context of their medical history, symptoms, and risk factors. The use of medical jargon and deviations from reference ranges may further contribute to stress, anxiety, confusion, and insecurity [9–11]. This potentially increases GPs' workload due to a higher demand for follow-up consultations to clarify results and discuss findings [11–13].

The emergence of online access is expected to affect doctor–patient communication in general and the communication of diagnostic test results in particular [8]. While face-to-face interaction remains fundamental to medical communication, maximizing the benefits of communicating via online portals requires improving their usability [14]. Previous studies have explored strategies to enhance patients' interpretation and comprehension of online laboratory and radiology test results [15, 16]. Findings suggest that reporting processes should shift toward a more patient-centered approach, emphasizing clarity, context, and presentation of diagnostic information to improve patients' information processing and make information more meaningful [15–17]. However, this shift requires insights into GPs' experiences in this evolving communication landscape, as they remain responsible for ordering, interpreting and conveying results, even when digital tools alter the timing, format, and content of communication [18, 19].

This qualitative study aimed to explore GPs' experiences and perceived challenges in communicating diagnostic test results to patients, particularly in the context of increasing online access.

## Methods

This article follows the Consolidated Criteria for Reporting Qualitative Research (COREQ) checklist and was approved by the Faculty of Health, Medicine and Life Sciences-Research Ethics Committee of Maastricht University (FHML-REC/2023/133).

### Design

We conducted a qualitative study using semi-structured interviews to explore GPs' experiences and challenges in communicating diagnostic information to patients. Thematic analysis was applied to identify key themes.

### Setting, participants, and recruitment

The study was conducted in Dutch general practice, where GPs act as patients' primary point of contact in the healthcare system. They provide continuous care, including ordering, interpreting, and communicating diagnostic tests [20]. This communication often occurs during time-limited consultations and increasingly through online patient portals [1]. Although nurse practitioners and doctor's assistants may also convey results, GPs and GPs in training are primarily responsible for the entire communication process, from test ordering to result communication. For this reason, only GPs and GPs in training were included. No specific exclusion criteria were applied.

Participants were purposively sampled to ensure variation in gender, age, years of work experience, and practice type. Recruitment occurred via the research team's professional network and LinkedIn. Interested GPs and GPs in training received study information covering purpose, time investment, confidentiality, and data storage. All participants provided verbal informed consent prior to the interview. Recruitment continued until data saturation was achieved.

### Data collection

A semi-structured interview guide was developed by the multidisciplinary team (Supplementary Appendix S1). It contained open-ended questions about (i) the current process of diagnostic test result communication, and (ii) perceived challenges when communicating diagnostic information, particularly in the context of patients' online access. The guide was pilot tested with qualitative researchers to ensure relevance and fluency of questions. Questions were refined after the third and sixth interview to improve clarity. No repeat interviews were conducted. Individual interviews were conducted between January and December 2024 by one of two female authors (F.M. or C.Z.), either in person at the participant's practice or online via Microsoft Teams. All interviews were audio-recorded and lasted on average 36 minutes (range 21–53 minutes). No field notes were taken during the interviews. Participants received a €25 voucher as reimbursement.

### Data analysis

Interviews were transcribed verbatim and coded using ATLAS.ti Web (version 25.0.1). Thematic analysis followed Braun and Clarke's framework [21]. Two authors (F.M. and C.Z.) independently coded transcripts and discussed discrepancies until consensus. Remaining disagreements were resolved with a third researcher (J.J. or A.D.). Through multiple rounds of discussion and reflection within the multidisciplinary research team, codes were grouped into overarching themes reflecting participants' perspectives. Representative quotations were selected to illustrate each theme. After 14 interviews, data saturation (covering both thematic and meaning saturation) was deemed reached, as no new codes or themes emerged and recurring explanations and interpretations for each code and theme were identified [22]. Three additional interviews were conducted to confirm saturation.

### Reflexivity

The research team consisted of a GP in training and PhD student with interest in online test result communication (F.M., MD), a senior medical student interested in primary care (C.Z.), a behavioral scientist with expertise in health communication (J.J., PhD), a practicing GP and professor with expertise in primary care diagnostics (J.C., MD, PhD), and a health scientist with expertise in co-creative research (A.D., PhD). To minimize researcher bias, the team engaged in bracketing throughout the study. Prior assumptions and professional perspectives were discussed at the start of the project and revisited during data collection and analysis. Regular interdisciplinary meetings were held to reflect on interpretations, challenge individual preconceptions, and ensure that emerging themes were grounded in the data rather than researchers' expectations.

## Results

Seventeen interviews were conducted with 18 participants, as one interview involved two GPs simultaneously. Participants had a mean age of 43 years, and the majority were practice owners (*n* = 14). One GP in training was included. Participants represented urban (*n* = 5), village (*n* = 10), and urbanized village (*n* = 3) practices across the Netherlands. All practices offered online access to test results, with patient use ranging from under 10% (*n* = 4) to over 50% (*n* = 3). Participant characteristics are summarized in Table 1.

**Table 1:** Characteristics of interview participants (*n* = 18).

GP	Sex	Age (in years)	Function	Working experience (in years)	Location of general practice	Patients’ use of online access (% of total patient population)
1	M	38	Practice owner	10	Village	>50%
2	F	42	Practice owner	14	Urbanized village	Unknown^b^
3	F	50	Practice owner	21	Urban	Unknown^b^
4	M	52	Practice owner	20	Village	<10%
5	F	48	Practice owner	18	Village	Unknown^b^
6^a^	F	44	Practice owner	16	Village	30%–50%
7^a^	F	47	Practice owner	17	Village	30%–50%
8	F	38	Locum	6	Urban	<10%
9	M	38	Practice owner	10	Village	<10%
10	M	40	Practice owner	10	Urban	<10%
11	M	43	Practice owner	11	Urban	30%–50%
12	F	30	GP in training	2	Urban	30%–50%
13	M	60	Practice owner	36	Urbanized village	>50%
14	F	45	Practice owner	15	Urbanized village	>50%
15	M	55	Locum	20	Village	Unknown^b^
16	F	34	Employment contract	8	Village	Unknown^b^
17	M	43	Practice owner	20	Village	Unknown^b^
18	M	35	Practice owner	9	Village	30%–50%

GP, general practitioner; M, male; F, female. ^a^Respondent 6 and 7 participated in the same interview. ^b^Exact percentage unknown, but online access was used by patients in general practice.

Themes were constructed from the data through an iterative and interpretive analytic process. The following were constructed: (i) managing patient expectations; (ii) purpose-driven communication strategies; and (iii) balancing efficiency and patient engagement in communicating test results.

### Theme 1: managing patient expectations

Communicating diagnostic information requires more than simply conveying test results, whether digitally or face-to-face. GPs highlighted the start of the diagnostic process, specifically when ordering tests, as a crucial moment for effective communication and management of patient expectations. By providing patients with clear and understandable information when tests are ordered, GPs aimed to simplify later result communication and reduce the need for lengthy explanations during follow-up consultations or in response to patient questions online or by phone.

*I think that if you clearly explain in advance what you’re requesting and why, you will need much fewer words later on. If you just say, ‘we are going to take a blood sample’ without any explanation, it is hard for the patient to understand what's going on afterwards.—Participant 1*

Within this communication process, two aspects were highlighted. First, GPs aimed to manage patient expectations by outlining possible scenarios in advance, thereby ensuring that patients are well-informed about the course of action when their test results are in. Second, GPs emphasized the importance of providing clarity to patients about when, how, and by whom their test results will be communicated.

*Uhm, well, I always discuss that. I also go over the possible scenarios—like what it means if there's a clear abnormality and what the next steps would be. But even more often, since that's statistically the most likely outcome, I discuss what we will do if the results don’t show any abnormalities.—Participant 17*

### Theme 2: purpose-driven communication strategies

GPs described three main purposes in communicating diagnostic information: (i) ensuring information is understandable for patients; (ii) stimulating behavioral change in patients; and (iii) providing reassurance to patients. GPs noted that they adapted their communication strategy to meet the intended purpose.

#### Ensuring information is understandable for patients

GPs expressed a strong sense of responsibility for offering clear and comprehensible explanations of diagnostic information. When results were conveyed through online portals, GPs emphasized the importance of providing additional clarification or interpretation tailored to the patient's context. To ensure effective communication, GPs highlighted the importance of using simple and unambiguous language, both in verbal and written communication. For example, GPs adjust their communication based on patient characteristics, including age, background, educational level, need for specific information, and patients' underlying care requests.

*Yes, yes, yes. I make sure not to use abbreviations. Uhm … I try to use lay language. Yes, really guiding the patient step by step, like ‘if this comes up, then we’ll consider that option, and here's why’. So sometimes that means a bit more typing on my part.—Participant 18*

#### Stimulating behavioral change in patients

GPs mentioned stimulating behavioral change in patients as an important communication goal. In cases where test results indicated a need for lifestyle changes, such as adopting a healthy diet or exercising more, GPs intentionally used communication strategies to encourage these behavioral changes. Both positive and negative framing techniques were mentioned to motivate patients to act on test results. While digital communication could initiate such encouragement, GPs favored face-to-face consultations to stimulate patient behavior change.

*For example, take a patient with diabetes—someone with poor values, like a high HbA1c or elevated glucose levels, who isn’t easily motivated to take action. In that case, you tend to frame things a bit more negatively, like, ‘this really isn’t good, we need to do something about this, we have to take action’. It's about getting the patient on board and helping them realize that something is genuinely wrong.—Participant 18*

#### Providing reassurance to patients

GPs emphasized providing reassurance as another communication goal. Reassurance was perceived as more easily achieved when there was an established doctor–patient relationship. Practice owners felt they were more successful in reassuring patients than when they worked as locum GPs, attributing this primarily to the greater trust patients typically place in their regular GP. While GPs did not associate patients' concerns with specific demographic characteristics, they did note that individuals with health anxiety or hypochondriacal tendencies required reassurance most frequently. Using positive framing was mentioned as a strategy to achieve reassurance, both in verbal and written communication.

*But even in those cases, I make use of the influence we have as general practitioners, you know. I might say something like, ‘just look at how many things we tested, everything came back great’. And that's where the social aspect comes in … As a doctor, you can … You don’t always have to justify everything with scientific precision. Sometimes, if it helps the patient, it's okay to frame things a bit more positively than they technically are. I mean, you could say, ‘the lab results are normal, but you could still have cancer—we can’t detect that with these tests'. That would be accurate. But you could also say, ‘if I were 80 years old, I’d be thrilled to have lab results like these—really great’. And I believe in that approach.—Participant 4*

GPs described occasionally using diagnostic tests as a communication and negotiation tool when verbal reassurance alone was insufficient. In these instances, the primary purpose of the test was not to confirm or rule out a medical condition, but to meet the patient's need for reassurance and to support the doctor–patient relationship by maintaining trust. This approach was particularly used when patients were initially unreceptive to the possibility of a benign explanation for their symptoms. The test result then helped facilitate future conversations about less serious causes and gradually aligned the patient's understanding with the GP's clinical perspective. In such cases, test results may be communicated either face-to-face or online, provided that possible outcomes and scenarios have been outlined in advance.

### Theme 3: balancing efficiency and patient engagement in communicating test results

GPs emphasized their need for efficiency in the healthcare process, especially given the increased workload in daily practice. Providing patients with access to their diagnostic test results online was often mentioned as an example of a more efficient and streamlined process, compared to delivering results during a face-to-face consultation.

*Well, one of the advantages is that I can go through everything in my own time and check things off more quickly. And it might not sound very sympathetic, but the less people talk back to me, the faster I can get things done. So, if I can handle it digitally—just give a quick summary like ‘everything's fine and here's the follow-up plan or next steps'—it takes way less time than when someone comes back with a bunch of questions. So that's definitely a big advantage.—Participant 17*

However, some GPs associated patients' online access to test results with a lack of efficiency in the healthcare process. Compared to situations in which GPs directly communicate test results to their patients, they noticed that online access often contributes to more uncertainty and questions. As a result, patients are more likely to initiate additional contacts to demand clarification of test results. GPs noted that addressing these concerns is time-consuming and they would rather spend that time on more urgent matters.

*But, of course, there are also patients who have lots of follow-up questions, like ‘what does this mean? What does this mean? What does this mean?’. And I think to myself, ‘it's just a value—something I’d normally just glance over’. But now I have to explain each value, and that takes time. And to be honest, I find that frustrating. It feels like time that isn’t helping the patient, except maybe by giving them a sense of control. I do understand that's valuable, and I get the importance of patient autonomy and knowledge. But in that moment, I’d rather spend my time giving a different kind of explanation.—Participant 2*

GPs identified several challenges related to patients' online access to test results. A key concern was that patients' lack of medical knowledge and experience can lead to misinterpretation of results, resulting in uncertainty, concerns, and questions. Additionally, GPs emphasized that online patient portals do not account for the individual context necessary for accurate interpretation of test results. They highlighted the importance of considering factors such as medical history, family history, reported symptoms, and potential risk factors. These contextual factors were typically integrated into result interpretation during clinical consultations but are absent in online result viewing unless added by the GP.

*It might be general advice, like, ‘it also depends on previous values, so you should take those into account too’. And as a doctor, you never just focus on one value. You always look at the whole picture: the symptoms, the value itself, and the patient's medical history.—Participant 11*

Concerns were particularly evident for online access to laboratory and radiology test results. One frequently cited issue involved clinically insignificant findings—results that appeared abnormal in the online portal but were considered clinically irrelevant or within normal limits by the GP. Examples included laboratory results flagged outside reference ranges and incidental findings in radiology reports. GPs noted that patients often interpret such findings as indicators of a health problem requiring action, while GPs do not consider them clinically concerning. Explaining these findings and providing reassurance was perceived time-consuming, particularly since GPs reported they would typically not discuss such findings in detail if patients had not accessed them online.

*That it's just fine. And that's the frustration, right? When patients take up our time … I find it frustrating when we get calls about lab results that patients can already view themselves. And it's hard to reassure them, because they see something like, ‘oh there's an asterisk next to this’, and then you end up explaining it three times.—Participant 10*

Another challenge was reported when patients accessed radiology reports. GPs noted that these narrative reports, written in complex medical terminology, are difficult for patients to understand and interpret without professional guidance. Consequently, GPs frequently receive patient requests for clarification or translation into lay terms, which they considered burdensome and time-consuming.

In contrast to laboratory or radiology results, GPs reported fewer difficulties in communicating microbiology results. This was primarily attributed to the typically straightforward nature of these results, with a pathogen either being present or absent, making them easier to explain. Moreover, GPs noted that microbiology results were often communicated to patients proactively due to clinical urgency. Consequently, patients tend to use online access for microbiology results less than for laboratory or radiology results.

GPs recommended using standardized text or disclaimers to support patients accessing online test results. They suggested that providing standardized additional diagnostic information in patient-friendly language, or disclaimers clarifying that not all abnormal values are clinically relevant, could help reassure patients and reduce unnecessary follow-up. This would help reduce GPs' workload and support their need for efficiency in the healthcare process.

*Now, reference values are published alongside the results, and you can see that a lot of things fall just outside those values. Ideally, you could maybe provide some explanation about… clinical relevance, right? And that it's still an interpretation by the doctor, and that something falling outside the reference range doesn’t necessarily mean a serious disease.—Participant 17*

## Discussion

This study explored GPs' perspectives on communicating diagnostic information in an era of online access for patients. GPs emphasized that timely communication, particularly when ordering tests, is crucial for managing patient understanding and expectations. By proactively outlining possible outcomes and planning follow-up steps, GPs aimed to enhance clarity and reduce confusion once results became available. This anticipatory approach supports GPs' broader goals of ensuring patient understanding, encouraging behavioral change, and providing reassurance. At the same time, the growing use of digital platforms to share diagnostic results with patients was seen as a double-edged sword by improving workflow efficiency for GPs but also prompting increased patient inquiries. GPs reported challenges including the misinterpretation of clinically insignificant findings and the complexity of medical jargon in radiology reports. This underscores the need for tailored communication strategies and potentially standardized explanatory tools.

### Comparison with literature

GPs emphasized the importance of managing patient expectations throughout the diagnostic process by providing clear explanations and discussing potential outcomes early on, aiming to reduce the need for extensive clarification later. This proactive approach aligns with findings from previous research highlighting the GPs' responsibility in supporting patients' understanding of test results, initiated ideally before a diagnostic test is ordered [8, 19, 23]. Early discussion of possible scenarios may reduce patient anxiety and fewer follow-up consultations [8, 19], enhancing patient engagement and mitigating diagnostic uncertainty [23, 24].

GPs mentioned three key purposes when communicating diagnostic test results to patients: (i) ensuring information is understandable; (ii) stimulating behavioral change; and (iii) providing reassurance. Stimulating behavioral change and providing reassurance were also reasons for ordering a diagnostic test in the first place. To achieve these goals, GPs reported using strategies such as patient-friendly language, personalized information, and positive or negative framing, in both verbal and written communication. These approaches are consistent with existing literature, which shows that patient-friendly language, visual aids, or personalized information can enhance patients' understanding and support positive health behaviors [25, 26]. Positive and negative framing have been recognized as effective persuasive strategies to encourage behavioral change, including smoking cessation [27], increased physical activity [28], and reduced alcohol consumption [29]. However, previous studies did not distinguish between online and face-to-face communication. Our findings indicate that when results require behavioral change, GPs prefer face-to-face communication to provide explanation and support.

While GPs occasionally used diagnostic tests to reassure patients, evidence suggests that diagnostic testing alone does not significantly enhance reassurance [30]. Instead, when diagnostic tests are clinically indicated, discussing potential scenarios and providing information about normal test results in advance is recommended [23, 30]. This proactive communication, whether verbal or digital, can contribute to patient reassurance, particularly when information is framed clearly, personalized, and contextualized.

GPs emphasized the importance of efficiency in communication, given increasing workloads. Online access to test results was perceived as both enhancing and hindering efficiency in the healthcare process, reflecting conflicting literature [8, 31]. On the one hand, online access can streamline the healthcare process by improving patients' knowledge and responsibility, thereby promoting patient engagement [8, 32]. Online communication is also considered less time-consuming compared to face-to-face consultations or phone calls [32, 33]. On the other hand, studies examining the broader use of online patient portals have highlighted GPs' concerns that patients may misinterpret medical information in general, causing confusion and anxiety [34, 35]. Additionally, online access has been associated with increased GP workload through more emails [32, 36, 37] and shows mixed effects on face-to-face consultations [13, 37].

The efficiency of communicating diagnostic results depends heavily on patients' ability to correctly interpret them, influencing understanding, emotional responses, and subsequent actions. GPs identified two main challenges in online communication: clinically insignificant findings and the complex medical terminology used in radiology reports. These findings are consistent with studies showing that both laboratory and radiology results are often difficult for patients to interpret without additional information or modification [15, 16]. A lack of medical knowledge increases the risk of assigning undue significance to minor abnormalities [15, 32, 34], while medical terminology in radiology reports further hinders comprehension [16, 38, 39]. These findings emphasize the need for patient-friendly presentation formats that rely on simple, unambiguous language.

GPs in our study highlighted their responsibility for supporting patient comprehension, often investing considerable time in providing explanations. Interestingly, their suggestion to use standardized text and disclaimers contrasts with their emphasis on providing context and previous recommendations for personalized information and illustrations to improve patients' information processing [15, 16]. Research on radiology reports underscores the delicate balance between specific, detailed information—which may validate patients but increase anxiety—and more general, non-specific information, which may reduce anxiety but foster confusion [40]. Considering this balance is crucial when communicating test results online. Witteman and Zikmund-Fisher formulated 10 recommendations to communicate laboratory results via online portals to support patient comprehension and usability [41]. Our findings align with several of their recommendations, including providing a clear message for each result, clarifying clinical relevance, and offering personalized information. Importantly, our study adds that face-to-face explanation prior to online communication can further enhance understanding and usability.

### Practical implications and future research

Insights from this study may inform the development and improvement of information presented in online patient portals, supporting more effective communication. We recommend initially focusing on laboratory and radiology results, addressing clinically insignificant findings and complex medical terminology. Providing additional information in clear, simple, and patient-friendly language is essential. Standardized text or disclaimers, explaining that not all abnormal results are clinically relevant, may help patients interpret their results more appropriately. At the same time, contextualization of test results will be necessary, especially when patients seek to understand what their results mean in their personal situation. Such personalized interpretation can help address individual concerns and make information more meaningful. Given GPs' need for efficiency, solutions should be designed to minimize additional workload.

Future research could focus on developing and testing additional information or modifications to online patient portals and evaluating their impact on both GPs' and patients' experiences, interpretations, and perceived challenges.

### Strengths and limitations

A multidisciplinary team of GPs, behavioral scientists, and health scientists contributed to this study, enriching data analysis and interpretation. Interviews were conducted and analyzed by two independent, medically trained researchers, which may have introduced researcher bias. However, several strategies were used to mitigate this risk, including bracketing discussions within the research team, the use of a semi-structured interview guide, and regular reflection on emerging interpretations. Recruitment through the team's professional network and LinkedIn may have introduced selection bias by favoring the inclusion of participants interested in doctor–patient communication and digitalization. However, variation in the extent of patients' use of online access was observed among included practices. Purposive sampling ensured variation in participants' sex, experience, and demographics. The inclusion of only one GP in training may underrepresent trainee perspectives. Other health care professionals involved in test result communication in general practice, such as nurse practitioners or doctor's assistants, were not included.

## Conclusions

Patients' growing online access to diagnostic test results requires a shift in interaction and communication between GPs and patients. This qualitative study provides insights into GPs' experiences and challenges when communicating diagnostic information. Findings can guide the development and improvement of online patient portals, aiming to enhance the efficiency of communication between GPs and patients.

## Supplementary Material

cmaf113_Supplementary_Data

## Data Availability

The data underlying this article will be shared on reasonable request to the corresponding author.
