# Supplementary material for: Balancing efficiency and misinterpretation: general practitioners' perspectives on communicating diagnostic test results in the digital era
Source: Fam Pract. 2026 Jan 13;43(1):cmaf113. doi: 10.1093/fampra/cmaf113 (PMC12798535; doi:10.1093/fampra/cmaf113)
Supplement: cmaf113_Supplementary_Data [file cmaf113_supplementary_data.zip › Appendix 1_interview guide.pdf]

Appendix 1. Interview guide

1. Demographics
    a. Age?
    b. Years of working experience as a medical doctor?
    c. Type of general practice, number of patients?
    d. Mean age and socioeconomic status of patients?
    e. Patients' use of online access to diagnostic test results in general practice?

Current situation: in preparation for this interview, we asked you to bring an example of providing diagnostic information to a patient. Could you walk me through this process; what do you say to the patient and when?

2. Practicalities
    a. How does the entire diagnostic process proceed in practice?
    b. Use of online access to test results?
    c. Use of additional tools/resources (images, documents, websites) when explaining results?
        i. If yes, are these additional tools effective?
        ii. If not, why not? What type of additional tools would you need?
    d. Are doctor's assistants involved in communicating diagnostic information to patients?
        i. Can all types of diagnostic information be communicated by doctor's assistants?
        ii. Do questions still end up with the general practitioner?
        iii. How do you know if information is correctly communicated to patients by doctor's assistants?

3. Patient's perspective

a. What are common questions arising from patients, related to diagnostic tests or test results?
b. What do patients mainly worry about?
    i. How do you notice?
    ii. How do you deal with this?

4. <u>Communication when requesting diagnostic tests</u>
    a. To what extent do you discuss the indication for a diagnostic test with a patient?
    b. What information do you provide in advance?
    c. Is there a difference in communication when diagnostics are used to prove a certain diagnosis versus when they are used to exclude a diagnosis?
    d. Is there a difference in communication of diagnostic information when monitoring versus performing a one-time diagnostic test?
    e. Use of expectation management?

5. <u>Communication when delivering diagnostic test results</u>
    a. How do you communicate diagnostic test results to patients?
    b. Do you experience a difference between delivering test results in person, by phone or digitally?
    c. Can you provide an example of your language when providing test results? Do you adjust your language?
    d. How do you determine whether to present information in a more positive or negative way to patients? Can you give an example of a situation where you chose positive or negative framing?
    e. How do you determine whether patients understood the information?
    f. Is it common for patients to view their test results online and still make an appointment with the general practitioner?

<u>Challenges in the communication process</u>

6. <u>Challenges faced by general practitioners</u>
    a. What challenges do you face when communicating diagnostic information to patients?
        i. How do you feel about the possibility for online access to test results?
        ii. On average, how much time do you spend discussing diagnostic information or results during a consultation?
    b. Can you give an example of a specific diagnostic test result that you often encounter challenges with?
        i. What makes this test result difficult to explain?
        ii. Do patients often have questions about this test?
    c. I will now focus on different types of tests. Can you indicate where you experience the most challenges communicating test results?
        i. Numerical (e.g. laboratory) versus textual (e.g. radiology) test results?
        ii. Laboratory test results.
            1. Which lab results cause the most difficulties explaining?
            2. Which lab results do patients have trouble understanding?
        iii. Radiology test results.
            1. Which radiology results cause the most difficulties explaining?
        iv. Medical microbiology test results.
            1. What about more difficult tests (e.g. STI tests, Borrelia), where timing of testing is important?
        v. Other diagnostic tests, such as spirometry and functional tests.
            1. What challenges do you face with these tests?

d. Can you provide an example of a situation where a patient expressed confusion about what a diagnostic test involved or why it was necessary?
    i. How did you notice this?
    ii. How did you address this at that time?
e. Can you provide an example of a situation in which requesting one diagnostic test leads to additional tests or the need to repeat tests (circular diagnostics)?
    i. How do you communicate about this?
    ii. How do you try to avoid this situation?
f. What are the advantages and disadvantages of online access to test results for you and the patient?
    i. Anxiety/concerns in patients?
    ii. Increase or decrease in number of consultations?
    iii. To what extent has online access to test results changed communication with patients?

7. <u>Patient characteristics</u>
    a. What type of patient tends to have more questions about their test results?
    b. What factors do you think influence this?
    c. Do you notice a difference between, for you, familiar and unfamiliar patients?

<u>Possible solutions</u>

8. <u>Possible solutions</u>
    a. When we start working on a solution for communicating diagnostic test results, what would be most helpful for you?
    b. What should we focus on first?

i. Which category (laboratory, radiology, medical microbiology) should be prioritized?

ii. Which specific tests within these categories should be prioritized?

c. How could simple and clear explanations of diagnostic information simplify the communication process?

  i. And at which stage or moment in the communication process would this be helpful?
